# Distributed 2D temperature sensing during nanoparticles assisted laser ablation by means of high-scattering fiber sensors

**DOI:** 10.1038/s41598-020-69384-2

**Published:** 2020-07-28

**Authors:** Zhannat Ashikbayeva, Arman Aitkulov, Madina Jelbuldina, Aizhan Issatayeva, Aidana Beisenova, Carlo Molardi, Paola Saccomandi, Wilfried Blanc, Vassilis J. Inglezakis, Daniele Tosi

**Affiliations:** 1 School of Engineering and Digital Sciences, Nazarbayev University, 53 Kabanbay Batyr Ave, 010000 Nur-Sultan, Kazakhstan; 2 PI National Laboratory Astana, Nazarbayev University, 53 Kabanbay Batyr Ave, 010000 Nur-Sultan, Kazakhstan; 3 Department of Chemical and Process Engineering, University of Strathclyde, 75 Montrose Street, Glasgow, G1 1XJ UK; 4 Department of Mechanical Engineering, Politecnico Di Milano, Via Giuseppe La Masa 1, 20156 Milan, Italy; 5 CNRS, INPHYNI, UMR 7010, Université Côte D’Azur, Parc Valrose, 06108 Nice, France

**Keywords:** Biophotonics, Optical metrology, Engineering, Materials science

## Abstract

The high demand in effective and minimally invasive cancer treatments, namely thermal ablation, leads to the demand for real-time multi-dimensional thermometry to evaluate the treatment effectiveness, which can be also assisted by the use of nanoparticles. We report the results of 20-nm gold and magnetic iron oxide nanoparticles-assisted laser ablation on a porcine liver phantom. The experimental set-up consisting of high-scattering nanoparticle-doped fibers was operated by means of a scattering–level multiplexing arrangement and interrogated via optical backscattered reflectometry, together with a solid-state laser diode operating at 980 nm. The multiplexed 2-dimensional fiber arrangement based on nanoparticle-doped fibers allowed an accurate superficial thermal map detected in real-time.

## Introduction

Laser ablation (LA) is a minimally invasive and alternative thermal therapy technique to the surgical resection for the treatment of cancer that aimed to destroy the tumor at high temperatures. Laser ablation works on the principle of conversion of absorbed laser light into heat in tissue leading to coagulative necrosis. The laser light inside the tumor is distributed according to the phenomena of scattering, absorption, and bending. The rate of absorption mainly depends on the water and hemoglobin load^1^. The widely employed laser types for LA are Neodymium: Yttrium aluminum garnet operating at a wavelength of 1,064 nm and solid-state diode working at a wavelength of 800–980 nm due to the best light penetration ability at near-infrared region^2^. However, currently, laser diodes gaining more interest and replacing the Nd: YAG laser because they are cheaper, easier to transport due to the lightweight and shows the similar tissue penetration performance at the wavelengths between 800 and 980 nm^3^.

In the last 4 decades, many studies have investigated the efficacy of LA for the treatment of cancer, demonstrated its advances, and evaluated the settings responsible for the therapy outcome. Indeed, the size of the heated area significantly depends on the laser power, laser wavelength, absorption features, optical properties of the biological tissue, and time of laser irradiation^4,5^. In the clinical practice, laser light is delivered in different modalities depending on the location of the tumor and the desired thermal effect: deep-seated tumors in the liver^6,7^, pancreas^8,9^, brain^10^ and other organs can be reached by thin optical applicators; superficial tumors like nonmelanoma skin cancer^11,12^, superficial bladder carcinoma^13^ can be treated with external and non-invasive approaches.

The pioneering work of laser application in tumor treatment was done by Bown in 1983^4^. LA is mostly known as interstitial laser thermotherapy, where the light delivered by means of delivery fiber into the tumor, usually a large core fiber or a side-firing fiber ranging in diameter from 0.5 to 2.5 mm^14–16^. The laser light is further converted into the thermal energy leading to the tumor damage. Nowadays, the reliable method of real-time imaging and the possibility to monitor the delivery of laser energy during LA is achieved through magnetic resonance tomography (MRI). MRI is working on the principle of dependency between temperature and proton resonance frequency^17,18^. However, the advantages of MRI monitoring is restricted by the difficulties in reaching the deep-seated tumor, and its radiation features, hard to transport the equipment and high cost^14^. To overcome these limitations the optical fibers with the smallest diameter of 100 µm can be an alternative to avoid the tissue damage, to obtain the accurate sensing technique and low cost^17^.

However, existing thermal ablation techniques including LA have restrictions in accurate temperature monitoring and can damage the surrounding healthy tissue around the tumor. The ability to have the precise thermal map and derive the optimal exposure time during LA can prevent the damage of healthy tissue. Nanoparticles combined with the ablation procedures are considered as a promising and advantageous approach to overcome these challenges because nanomaterials can selectively and specifically bind to the targeted region and increase the heating temperature^19^.

Nanoparticles are materials of size between 10 and 100 nm, which offer beneficial optical and magnetic properties. Nanoparticles are able to elevate the heat by absorbing the near-infrared light, and introducing in situ a high density of scattering centers, that randomizes the light path and help to spread the heat towards peripheral sides of the tumor, hence increasing the treated region. The efficacy of nanoparticles’ use during thermal therapy has been proven and discussed for many years. The most used metallic nanoparticles are magnetic iron oxide and gold nanoparticles due to their unique properties, biocompatibility, and ease of synthesis^20^.

Fiber optic sensors (FOS) are promising alternatives for accurate temperature monitoring and shape sensing compared to conventional methods such as Magnetic Resonance Imaging (MRI) and Computed Tomography (CT) sensing methods. MRI and CT are the most used imaging technique used during the ablation process^14^. Optical fibers offer distinctive properties due to their biocompatibility (ISO 10993 standard), small size, and ability to detect the change of temperature, pressure, or strain^21^. Moreover, optical fibers are inert to chemicals and electromagnetic interference because of their non-metallic composition as fibers are made of fused silica^22^. There are two known approaches for temperature sensing with high spatial resolution. The first technique employs the Fiber Bragg Grating (FBG) arrays where several sensors multiplexed in a single fiber and employing the wavelength division multiplexing^23^. However, this approach has some drawbacks due to the distance between FBGs leading to a decline in the accuracy of temperature change^24^. A second approach is distributed sensing, working on the principle of frequency domain reflectometry: optical backscattered reflectometry (OBR) is the most common implementation of this approach, and it is implemented by introducing the Rayleigh scattering patterns of a sensing fiber^25,26^. The OBR achieves a millimeter-scale spatial resolution, with a trade-off between accuracy and resolution better than FBGs^27^.

The purpose of our study is to provide a significant improvement in LA in situ thermometry, by improving the distribution feature of the OBR sensor. For this reason, we employ a configuration labeled scattering—level multiplexing (SLMux)^28^, which makes use of high-scattering fibers doped with manganese oxide nanoparticles in the core. The set up arranged from three parts providing the key advance in temperature sensing approach when working together: (1) the solid-state laser for ablation, coupled in its delivery fiber; (2) two types nanoparticles and their solvent; (3) the fiber optic sensing network; and thanks to the different type of NP we can estimate the effect on the ablation outcome. With this configuration, an accurate temperature distributed sensing during the laser ablation in 2-dimensions using four fibers, each operating in distributed sensing mode can be monitored, and thanks to gold and iron oxide magnetic nanoparticles the estimation of its effect on the ablation outcome was obtained.

This work for the first time presents the damaged/treated-area analysis during nanoparticle-mediated laser ablation, which evaluates the advantages and impact of the proposed method in achieving 2D thermal ablation performance and temperature pattern monitoring in real-time. Noteworthy that the configuration of sensing network SLMux allowed obtaining a high spatial resolution of 0.3 cm that is comparable to MRI, making it possible to monitor any change of the temperature distribution in tissue precisely. Moreover, this study demonstrated a good heating pattern during LA when nanoparticles were used.

## Materials and methods

### Experimental setup

**Figure 1:**
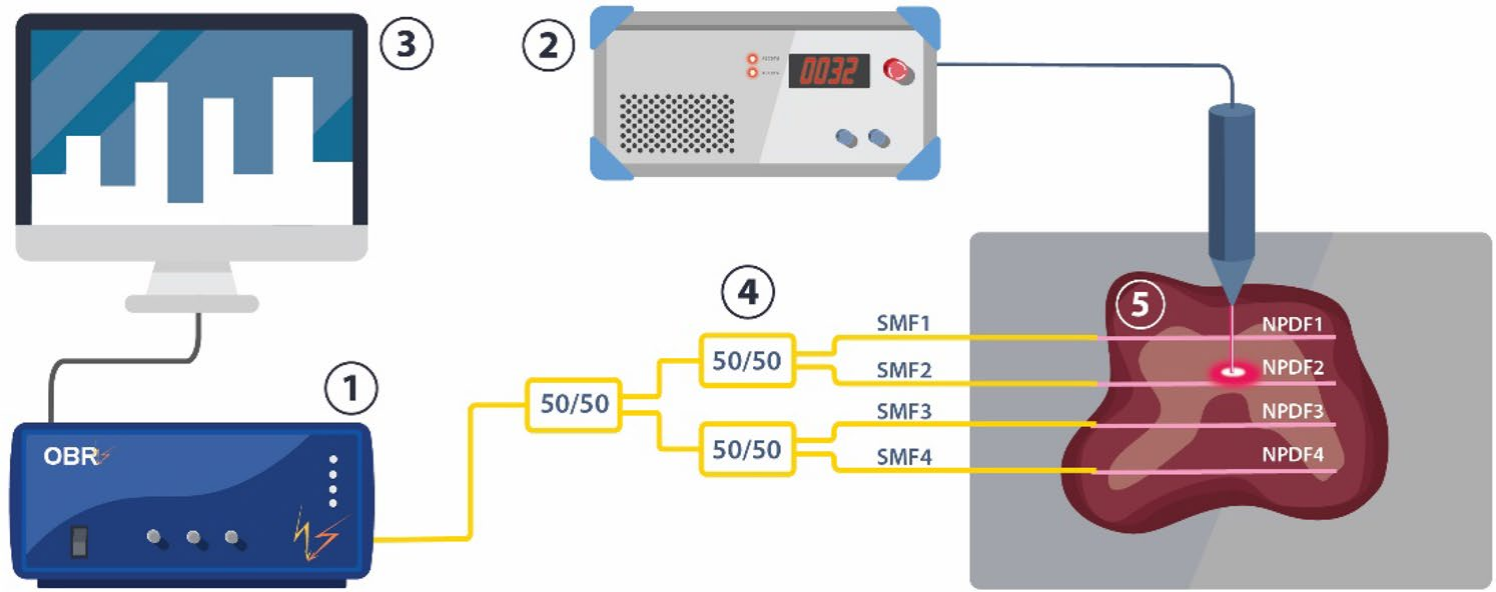
Experimental set up for laser ablation of the porcine liver: (1) optical backscattering reflectometer (OBR), (2) fiber-coupled laser diode, (3) data processing software for OBR and laser, (4) set of four NPDF optical fibers, connected to single-mode fibers, where yellow-colored fiber is a single-mode fiber (SMF) and pink-colored fiber is NPDF, (5) porcine liver phantom.

**Figure 2:**
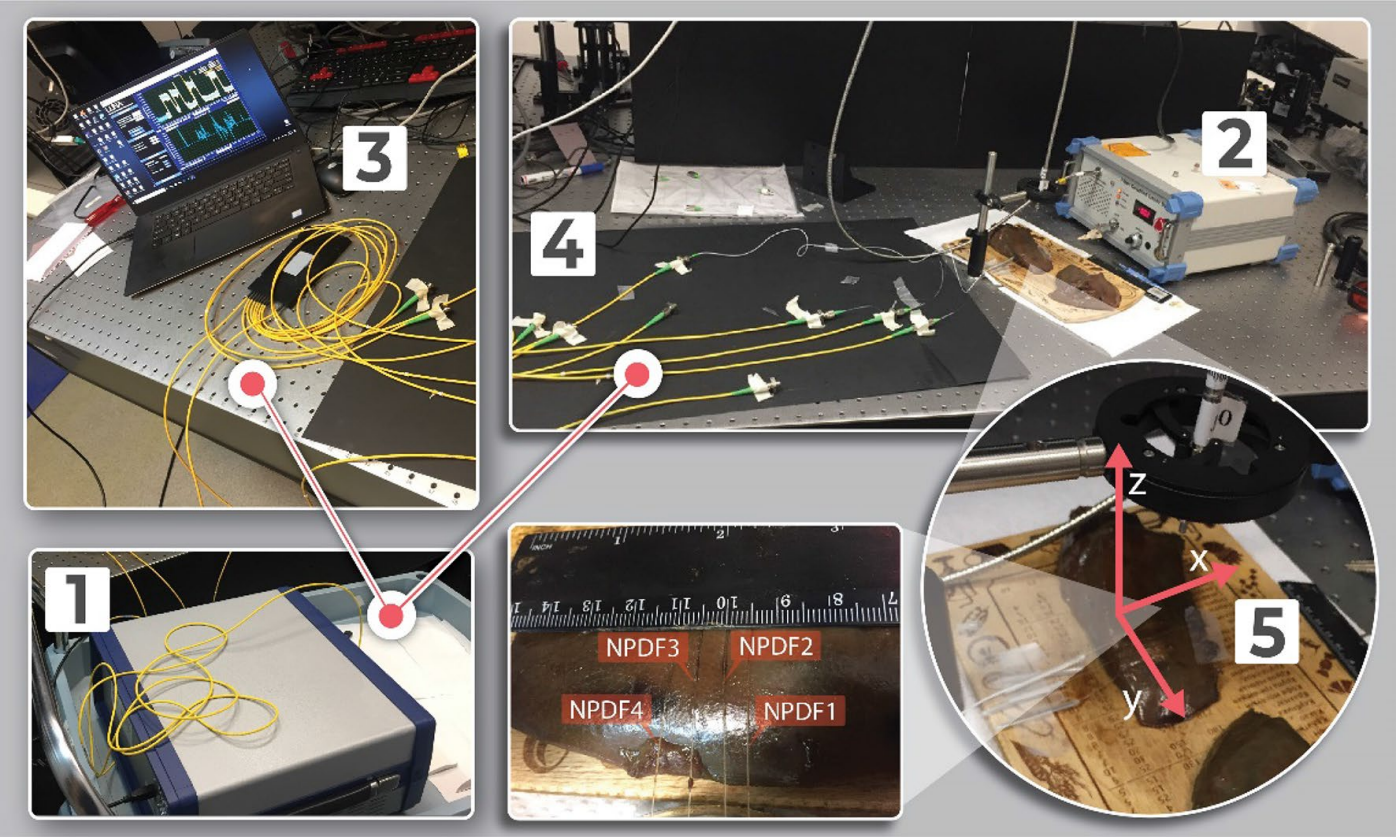
Photograph of the experimental set-up for nanoparticle assisted laser ablation of the porcine liver. (see Fig. 1 for numbering of devices). The figure allows seeing the placement of NPDF-fibers in tissue at a distance of 5 mm along xy-plane.

The experimental set up of multiplexed temperature monitoring during laser ablation of the porcine liver phantom using nanoparticles gold and magnetic iron oxide is represented schematically in Fig. 1, with the photographic view in Fig. 2. The sensing design consists of: (1) an Optical backscattering reflectometry (OBR), (2) fiber-coupled laser diode, (3) data processing software, (4) four nanoparticle doped optical fibers (NPDF), (5) fresh porcine liver.

The ablation procedure was conducted by means of fiber-coupled mid-power laser operating in a continuous mode (980 nm, Roithner Lasertechnik GmbH, Austria). The laser was calibrated and set at a current of 2.34 A which corresponding to 4 W and the ablation procedure lasted from 120 to 145 s. The laser light was delivered to the liver phantom through 1 m long fiber applicator with the core diameter 400 µm and numerical aperture 0.22 at a 75 mm distance in the z-direction (Fig. 2). The delivery fiber is clearly multimode and emits with a very large divergence angle θ. The beam waist can be approximated to the radius of the fiber 200 µm. The divergence angle θ is related to the aperture number NA with the well know relation: sinθ = NA. The spot size radius can then be estimated as:

$$0.2\;{\text{mm}} + 75\;{\text{mm}} \times \tan\uptheta = 0.2\;{\text{mm}} + 75\,{\text{mm}} \times {\text{NA/}}\surd \left( {1 + {\text{ NA}}^{2} } \right) = 17.1\;{\text{mm}}$$

This is an approximated assumption, however, it is similar to the laser ablation setup with frontal firing fibers (as in the example Table 3 of ref.^3^).

The laser applicator was fixed using a holder on a perpendicular direction to the tissue as shown in Fig. 2 to conduct the laser ablation on the tissue surface. The thermal ablation process was done following the European protocol of “Three Rs”^29^ on a commercially available porcine liver purchased from the butchery shop and maintained at a room temperature 22–25 °C right prior to the experiments. The liver phantom stabilized until room temperature for several hours in order to mimic a real application close to the body temperature. The initial temperature was monitored using contact thermocouple (IKA ETS – D5). Moreover, a FLIR thermal camera was used to validate that the tissue temperature distribution is uniform, at least within the accuracy of the thermal camera. The accuracy of all FLIR cameras for IR detection at temperatures 0–120 °C ranges from ± 2 to ± 3 °C.

**Figure 3:**
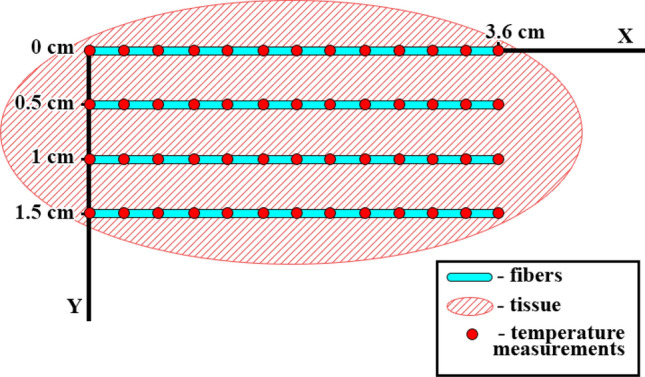
Schematic alignment of the fibers on the tissue.

Temperature measurements are performed with distributed fiber optic-based sensing setup, consisting of commercial OBR (OBR4600, Luna Inc.) to interrogate spectra of the fiber parallel. The fiber parallel is made up of 4 SMF-28 extenders each spliced to a piece of NP-doped fiber using the Fujikura 12-S fusion splicer. A 1 × 8 splitter is used to create 4 lines of the parallel in the Y direction. Distributed temperature measurements taken at longitudinal points on the fibers were used to fill the 2D-map as shown in Fig. 3.

In this setup, temperature changes are measured by analyzing the backscattering profiles of NP-doped fibers, while SMF serves as connecting elements. The backscattering profile is shifted in response to a temperature change so that it can be determined by comparing the measured spectra with the initial, or reference trace. It is important, that backscattering profiles of each of 4 NP-doped fibers do not overlap; to prevent such overlapping the length of each SMF-28 separator must be longer than the previous.

**Figure 4:**
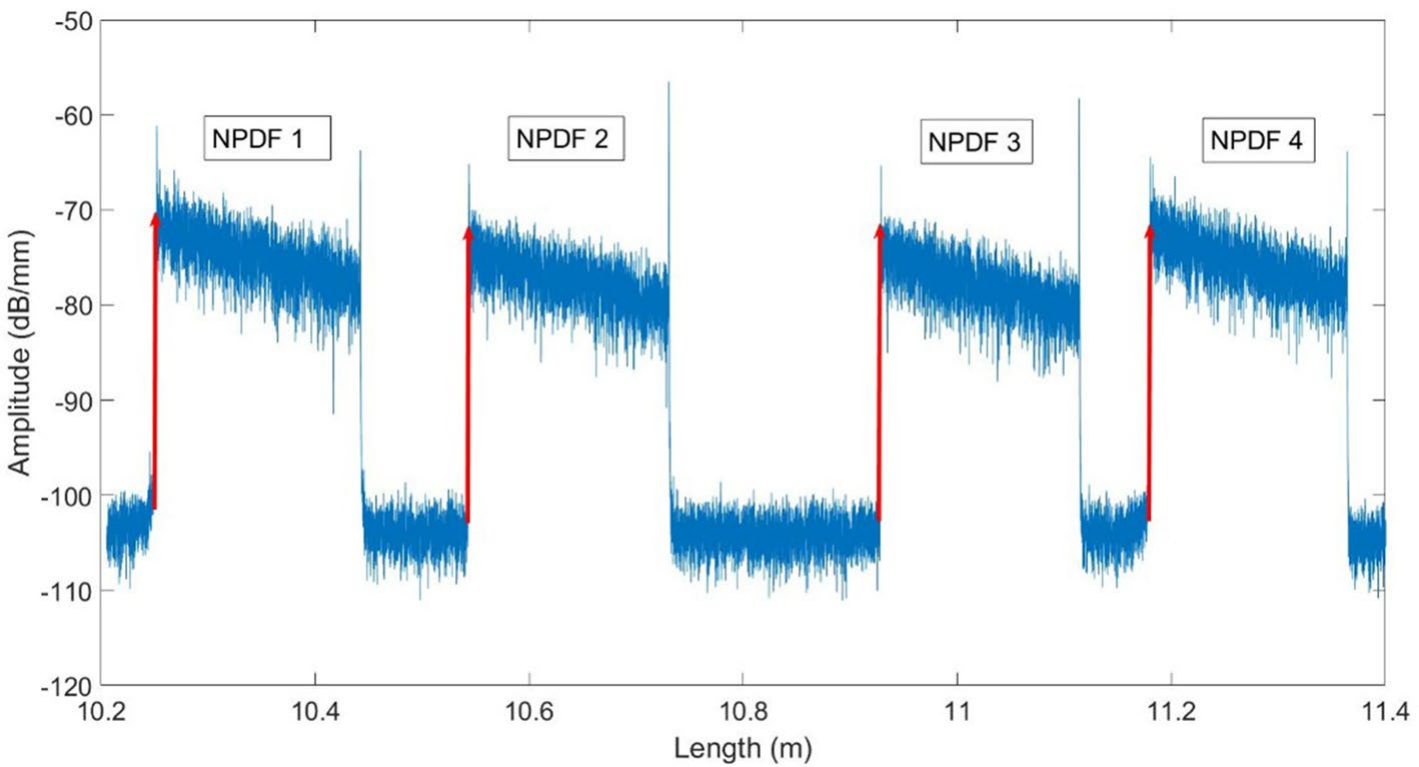
Backscattered trace of the sensing network, reporting the backscattered signal amplitude on the OBR at each length. The chart shows the contribution of the 4 fibers clearly visible over the SMF contributions.

OBR based on the principles of Optical Frequency Domain Reflectometry, which allows employing single-mode fiber as a valuable spatially distributed temperature sensor^24^. OBR has a great advantage for the thermal ablation application due to its high resolution (less than 1 cm)^21^. However, a serious limitation of OBR is its inability to provide multiple sensing fibers. Thermal ablation requires spatial two-dimensional temperature measurement, which cannot be achieved by a single fiber. Therefore, in this experimental set up we used nanoparticles-doped fibers (NPDF), which have about 30 dB higher scattering level than the standard single-mode fibers (SMF-28) as shown in Fig. 4. The NPDF fiber has been fabricated in two steps: at first, using modified chemical vapor deposition (MCVD) a silica preform was fabricated, with the addition of MgCl_2_ and ErCl_3_ dopants; then, the fiber was drawn in a standard drawing tower. The high temperature up to 2000 °C causes a separation of silica and alkaline ions, forming MgO-based nanoparticles with 20–100 nm diameter that elongate along with the fiber through the drawing process^30,31^. Four NPDFs with the core diameter of 10 µm and 125 cladding diameter are spliced with SMF pigtails of different lengths so that two neighboring NPDFs are spatially separated by the SMF fiber from the neighboring NPDF. The presence of MgO NP induced a much larger Rayleigh scattering. The spectrum, illustrated in Fig. 4, shows that the desired multiplexing is achieved because NPDFs are separated by the regions of lower scattering power of SMF^28^.

The 4 NPDF sensing fibers have been positioned on the liver porcine tissue in situ at a distance 0.5 cm that can be seen from photographic view in Fig. 2 from each other in y-direction. Such positioning allows monitoring the changes in temperature not only at the center of the ablation area but also in the peripheral area. Figure 3 shows graphically the positions of the fibers with respect to the tissue: the lines report the 4 fibers, and the dots the sensing points along the fibers detected by the OBR. The temperature sensing technique consists of 52 sensing points over the 5.4 cm^2^ area.

All trials were performed at the fixed settings of laser and optical fiber sensing parameters with the in situ positioning of fibers on the tissue. However, the only changing condition during the LA was the treatment of the tissue with nanoparticles and without nanoparticles. In addition, two different types of metallic nanoparticles were employed, namely gold and magnetic iron oxide nanoparticles. Both nanoparticles were spherical in shape and had a size of around 20 nm. Magnetic iron oxide nanoparticle was diluted in a 0.2% agarose solution with a concentration of 5 mg/ml. The aqueous stock solution of gold nanoparticles with a concentration of 0.055 mg/ml was used in the ablation procedure. The 100 µl of nanoparticle solution (both gold and magnetite nanoparticles) from previously prepared stock were injected ex vivo onto the liver phantom using the pipette. This setup, having a short distance between the fiber output and the tissue, has been designed to mimic noninvasive and superficial laser ablation^11,13^.

### Data acquisition and analysis

Temperature values were collected along the lengths of the fibers by the optical backscatter reflectometer operated in a distributed sensing mode. The step size between each temperature measurement was 0.3 cm. Spaces between each pair of the fibers were 0.5 cm, and the length of each fiber segment under measurement was 3.6 cm. The alignment of the fibers is schematically represented in Fig. 3.

Data acquisition was conducted at a rate of one measurement/s. Then, the resulting temperature measurements were interpolated with the spline method, introducing additional data points at intervals of 0.02 cm across the XY-plane in Fig. 3. The ‘pixel size’ is 3 mm (X) × 5 mm (Y), so 15 mm^2^ areas over a surface of 36 mm ×15 mm = 540 mm^2^. The 2D fit interpolates the data on a grid of 0.2 mm × 0.2 mm = 0.04 mm^2^ (13,500 interpolated sensing points).

Each value of temperature was then assigned a specific color. The resulting 2D colored maps were saved for all points in time throughout each experiment on ablation. In addition, quiver plots were produced for all of the thermal maps to clearly indicate the direction of temperature change in the experiments.

### Fiber calibration

**Figure 5:**
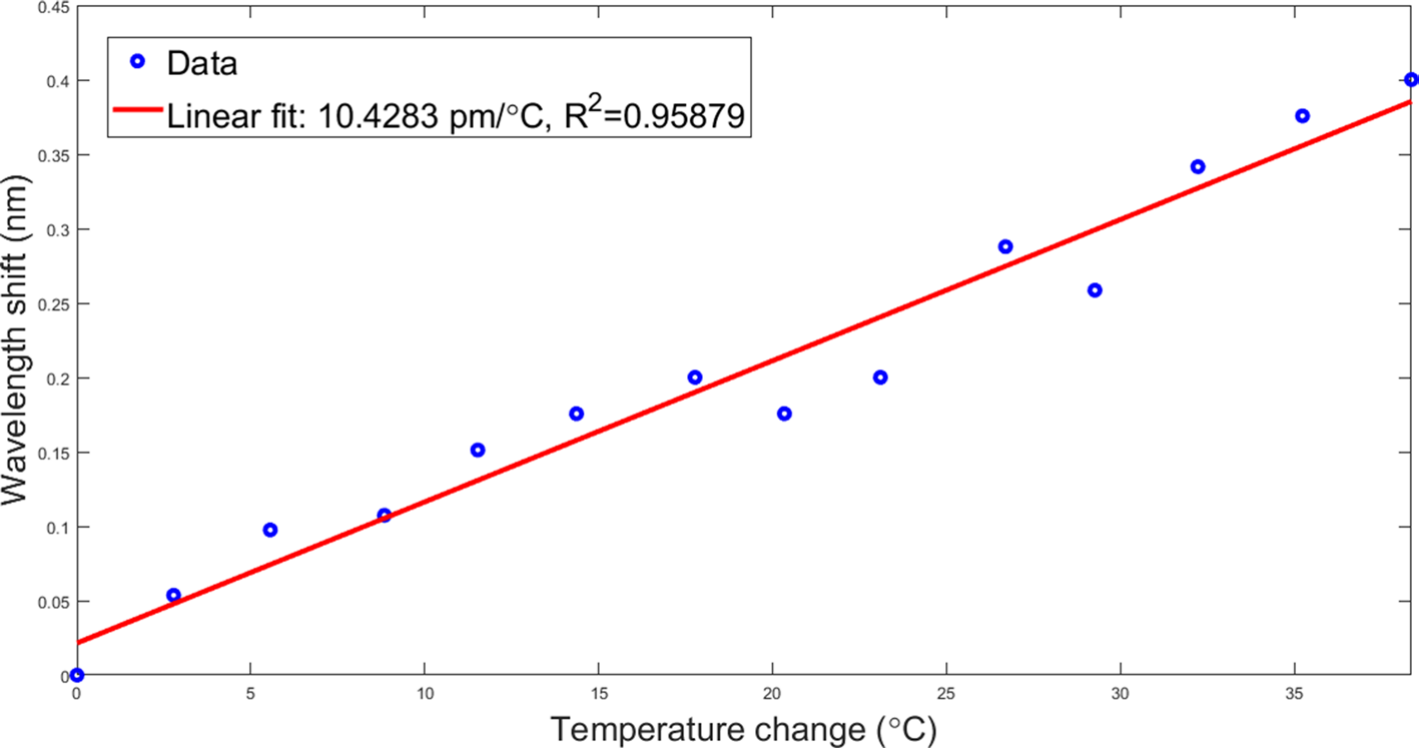
Wavelength shifts with respect to temperature changes as recorded by NPDF fibers.

Four NPDF fibers used in the experiments were thermally calibrated first. They were immersed in a flask filled with water. A fiber containing one FBG (Technica S.A., 10.2 pm/°C) was also placed there to provide a reference. The water inside the flask was heated while monitoring the spectra recorded by the 4 fibers and the FBG. Temperature changes were calculated using the wavelength shifts of the FBG. Then, temperature changes indicated by the FBG were compared with the shifts recorded by four fibers. The results, including the sensitivities of all fibers, can be seen in Fig. 5. According to the calibration results, all the fibers have the same sensitivity coefficient, which is equal to 10.42 pm/°C and is similar to FBGs written on standard glass fibers.

### Synthesis and characterization of nanoparticles

#### Gold nanoparticles

The synthesis of gold nanoparticles was conducted in a three-necked 250 mL round-bottomed flask cleaned with a dichromate solution according to the method of^32^. The set up was placed on a hot plate fitted with a magnetic bar for continuous stirring in the silicon oil heating condition. 0.5 ml of 1% stock solution of hydrogen tetrachloroaurate (III) trihydrate first added into the flask through funnel followed by 50 ml of distilled water (DI). The hot plate adjusted to the temperature of 105–108 °C. The resulting solution was heated until boiling. Afterward, 2.0 ml of 34 mM citrate solution was added to the solution. The solution was refluxed further for 15 min under stirring conditions and slowly cooled down to room temperature under stirring. Finally, the obtained gold nanoparticles were washed two times with DI water using a centrifuge for 15 min under 12,000 rpm and stored in vials at room temperature. The color of the final nanoparticle solution was ruby-red. UV–VIS spectral analysis and TEM scanning were performed for each set of experiments. All chemicals were of analytical grade and purchased from the Merk Company.

**Figure 6:**
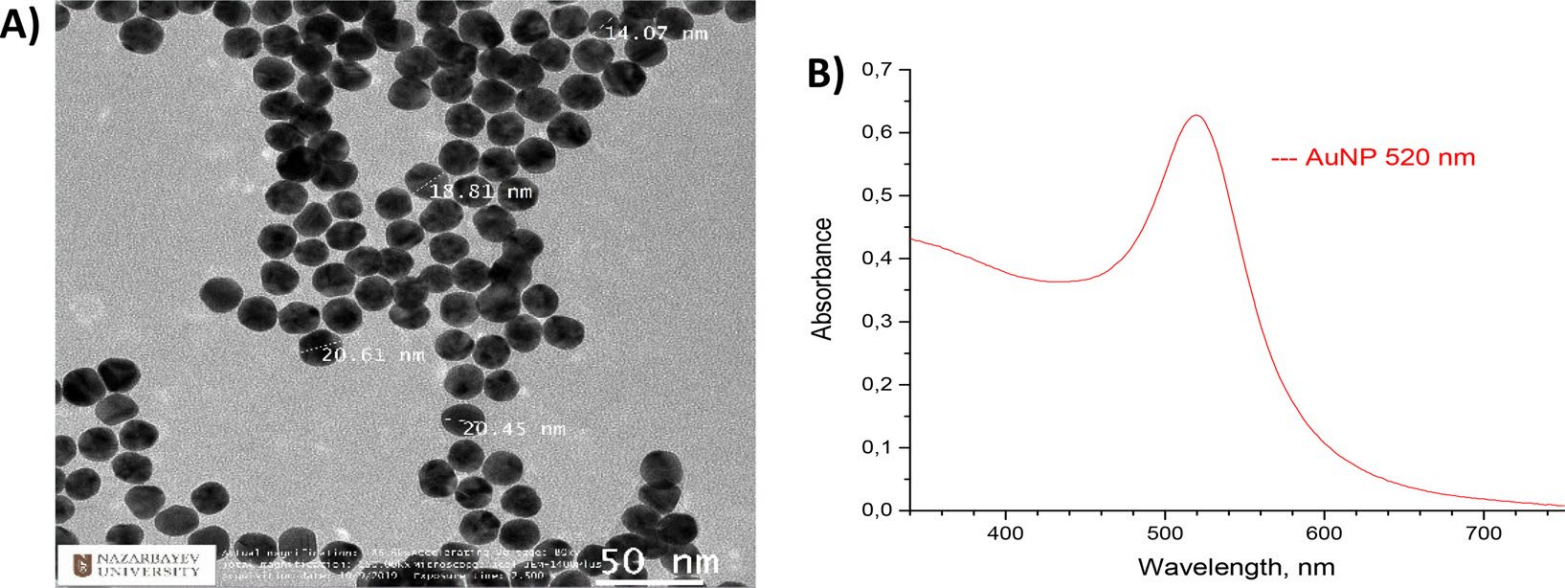
(**A**) TEM micrographs of 20 nm synthesized gold nanoparticles obtained by JEOL JEM—1,400 Plus microscope. (**B**) Absorption spectra of synthesized gold nanoparticles obtained by UV–VIS spectral analysis.

For TEM analysis the samples were prepared by dropping the aqueous solution of gold nanoparticle solution on 400 copper mesh and left for drying at room temperature. The samples were analyzed on Transmission Electron Microscope (JEOL JEM—1400 Plus). The obtained TEM micrographs of gold nanoparticles demonstrate the homogenous distribution of the nanoparticles with the size range between 14 and 20 nm. The shape of the gold nanoparticles is spherical according to the obtained data in Fig. 6a.

Figure 6b shows the absorption spectra of synthesized gold nanoparticles analyzed on Evolution 300 UV–Vis Spectrophotometer. The absorption maximum of AuNPs was 520 nm, which stands for nanoparticles of size 20 nm^33^. This is in agreement with the expected ruby-red color of the solution, which also indicates that the size of nanoparticles is 20 nm.

#### Magnetic iron oxide magnetic nanoparticles

The iron oxide magnetic nanoparticles were synthesized by the solvothermal method adapted from the method^34^. 2.535 g of FeCl_3_*6H_2_O and 1.8625 g of FeCl_2_*4H_2_O were dissolved in 6.25 ml of distilled water under the magnetic stirring in a beaker followed by the addition of 6.25 ml of 25% Ammonium Hydroxide. The obtained solution was stirred at 700 rpm for 2 min. Then placed into the Teflon-lined stainless steel autoclave and heated for 1 h at 180 °C in a muffle furnace. The synthesized nanoparticles were washed several times with distilled water and dried for further characterization and application for the thermal ablation. The X-Ray Diffraction (XRD) and Scanning Electron Microscopy (SEM) analysis were done in order to validate the size and shape of nanoparticles. All chemicals were purchased from Merk Company. The use of TEM analysis was avoided for magnetite due to its magnetic property.

**Figure 7:**
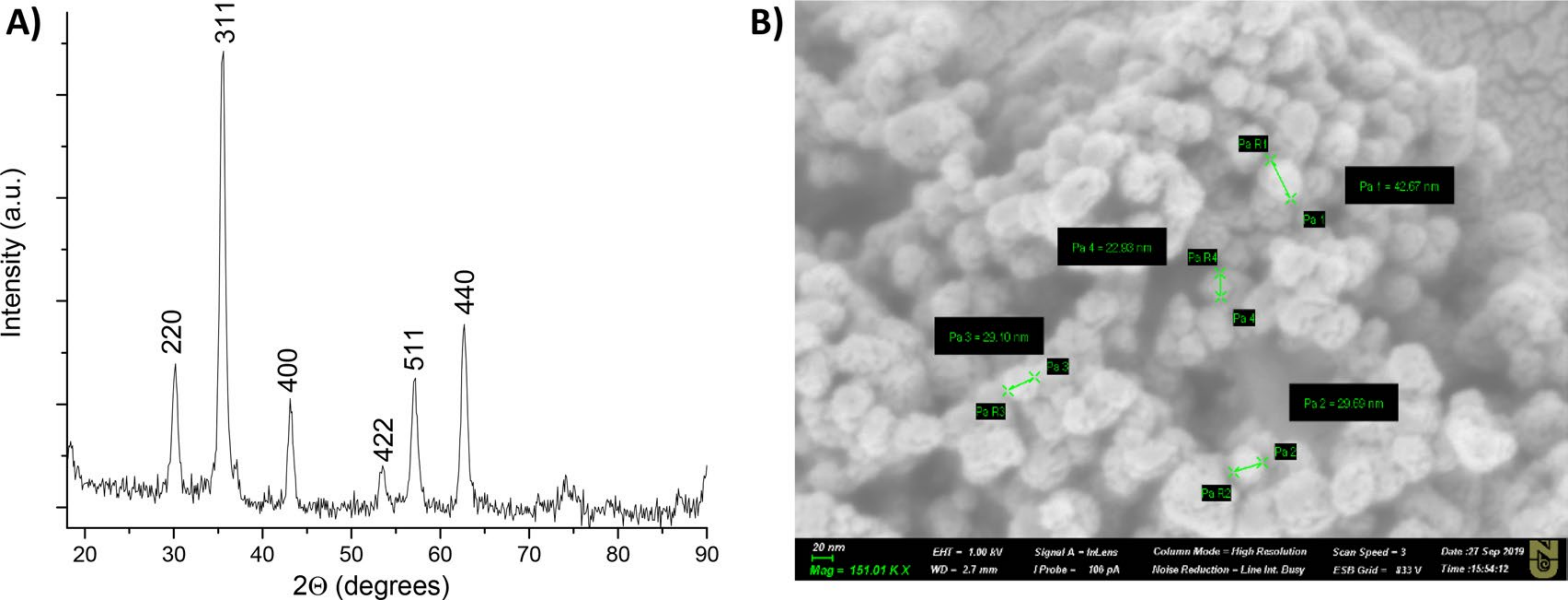
(**A**) XRD analysis data of 20 nm iron oxide nanoparticles. (**B**) The SEM image of 20 nm iron oxide nanoparticles obtained by Auriga Crossbeam 540 Microscope at the 151.01 KX magnitude.

The crystal structure and the size of the synthesized Fe_3_O_4_ nanoparticles were analyzed by the Rigaku SmartLab X-ray diffraction (XRD) system in Fig. 7a. The diffraction peaks obtained at 30.1°, 35.4°, 43°, 53.4°, 56.9°, 62.5° meet the diffractions values of [220], [311], [400], [422], [511] and [440] planes of Fe_3_O_4_ crystals respectively. The obtained XRD patterns matched the literature value for magnetite peaks^35,36^.

The size of the iron oxide magnetite was calculated as 20 nm using Scherrer’s equation. While, the surface morphology of iron oxide nanoparticles was observed using Scanning Electron Microscope (SEM, Auriga Crossbeam 540), which shows mostly uniform distribution with the size around 20–40 nm as demonstrated in Fig. 7b.

## Results

The experimental results were collected from combining thermal measurements registered by OBR for all of the four fibers in the scanning range between 1525 and 1611 nm. The 2D thermal map captured on the x–y plane analyzing the temporal data attained from 4 fibers, where the resolution along the y-axis was 0.5 cm and along the x-axis was 0.25 cm. Several experiments were performed to show the significance of the results. The experiments varied by the treatment of tissue with magnetic iron oxide nanoparticles and gold nanoparticles.

**Figure 8:**
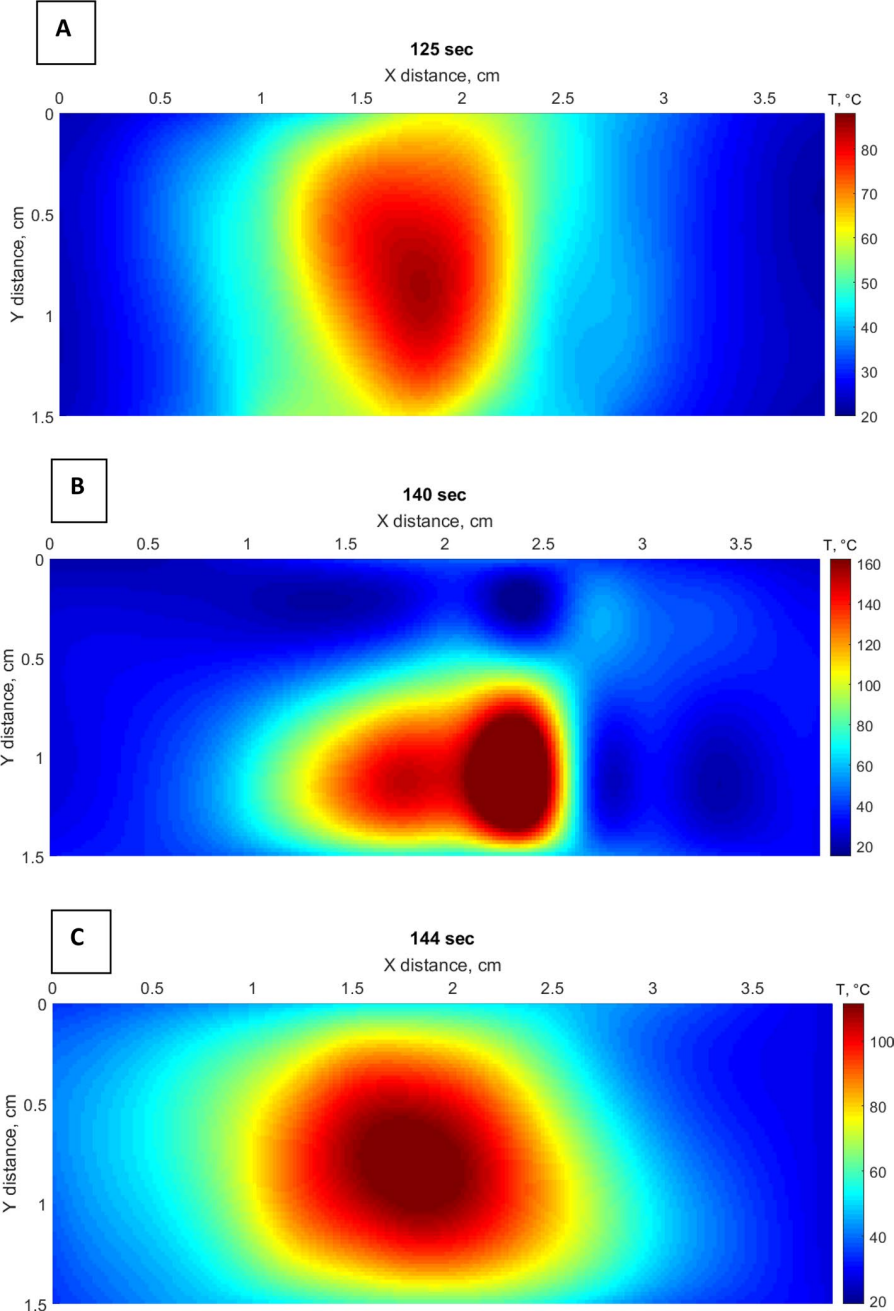
2D thermal of distributed sensing by 4 NPDF fibers in x-direction obtained by combining the values from all fibers for tissue: (**A**) without any nanoparticles treatment; (**B**) treated with 20 nm magnetic iron oxide nanoparticles; (**C**) 20 nm gold nanoparticles.

We show a 2D x–y temperature pattern for each experiment (Fig. 8a–c) that demonstrates considerable heat increase during the laser ablation when nanomaterials were employed. It is noteworthy that magnetic iron oxide nanoparticles exhibited higher temperature enlargement compared to gold nanoparticles with a maximum recorded temperature of 160 °C in 140 s at the center of the treated region. However, gold nanoparticles demonstrated symmetrical round shape and a bigger ablation area of 2.57 cm^2^ at 60 °C as compared to other ablation procedures that are presented in Table 1.

**Table 1:** The ablation area during LA at two temperature points: 42 °C and 60 °C.

Tissue	Heated area at 42 °C (cm^2^)	Heated area at 60 °C (cm^2^)	Max. temperature (°C)
Tissue without nanoparticles treatment	2.66	1.33	88.11
Tissue treated with iron oxide nanoparticles	2.53	1.60	162.39
Tissue treated with gold nanoparticles	4.34	2.57	111.60

### Temporal evaluation

**Figure 9:**
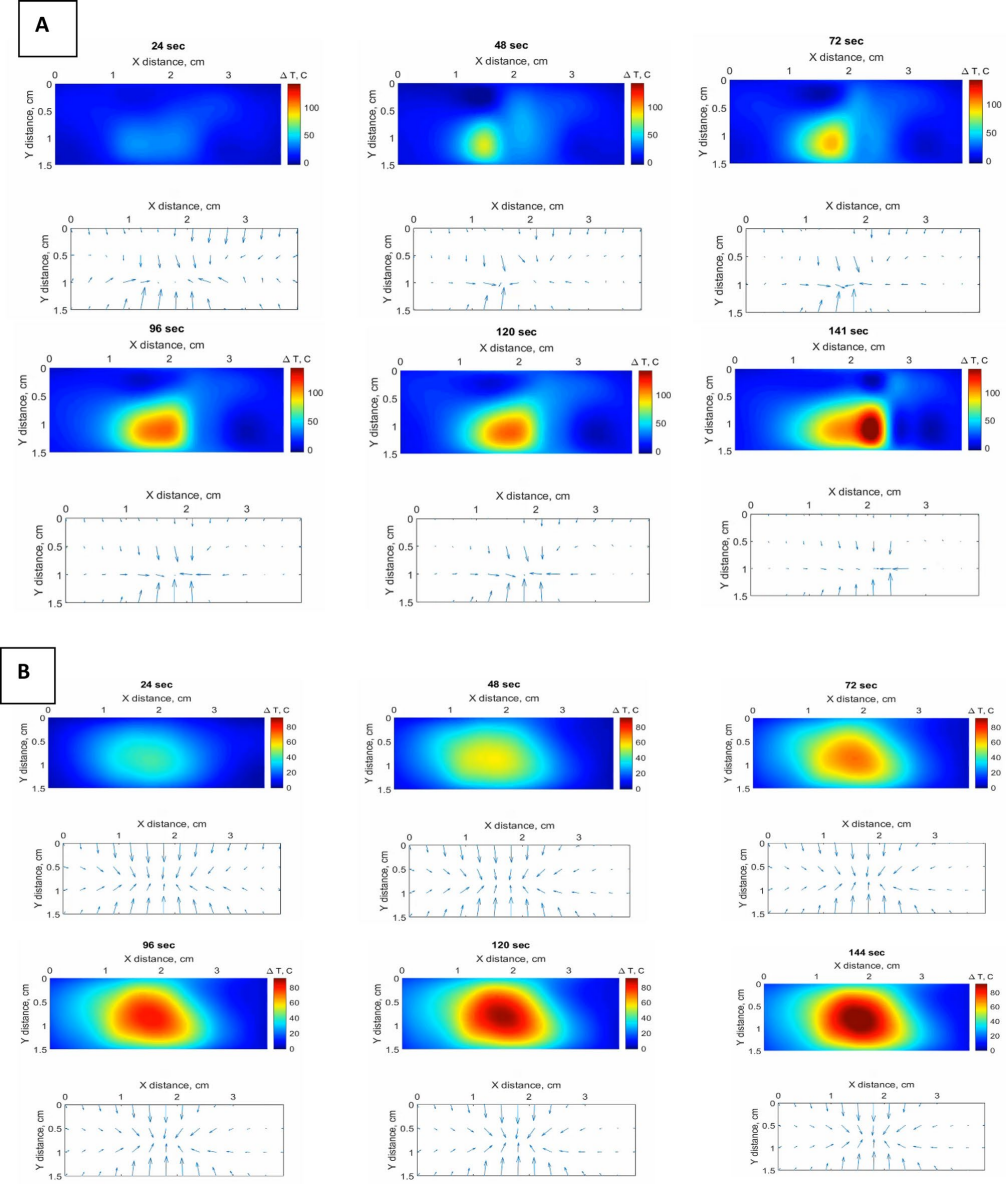
Cinematic view of temperature change at 6-time stamps for LA, reporting the XY temperature map, using (**A**) magnetic iron oxide nanoparticles; (**B**) gold nanoparticles.

The cinematic view of temperature increment monitoring during the LA is presented in Fig. 9a,b to show how the temperature changed over the ablation procedure. The 2D thermal map for the XY plane at 24, 48, 72, 96, 120, and 141 s was captured to display the temperature change. The results demonstrate that gold nanoparticles perform symmetrical spherically distributed temperature increases. The video recorded during the ablation is presented in Supplementary materials.

The temporal evaluation helps to determine the threshold temperature. The possibility of temporal evaluation in real-time allows us to determine when we need to terminate the ablation process because we do not want to measure the temperature at the end of the whole experiment. The proposed method is transferable for further implementation in situ for thermal therapies. The gradient plot shows the different heating process, highlighting the difference in the nanoparticle performance: during the gold nanoparticle-mediated experiment, the gradient has an almost constant shape, both in amplitude and direction of the thermal field; the iron oxide nanoparticle-mediated experiment shows a progressive deviation of the thermal field from the initial configuration, with a significant modification of the heating process.

### Cytotoxicity regions

**Figure 10:**
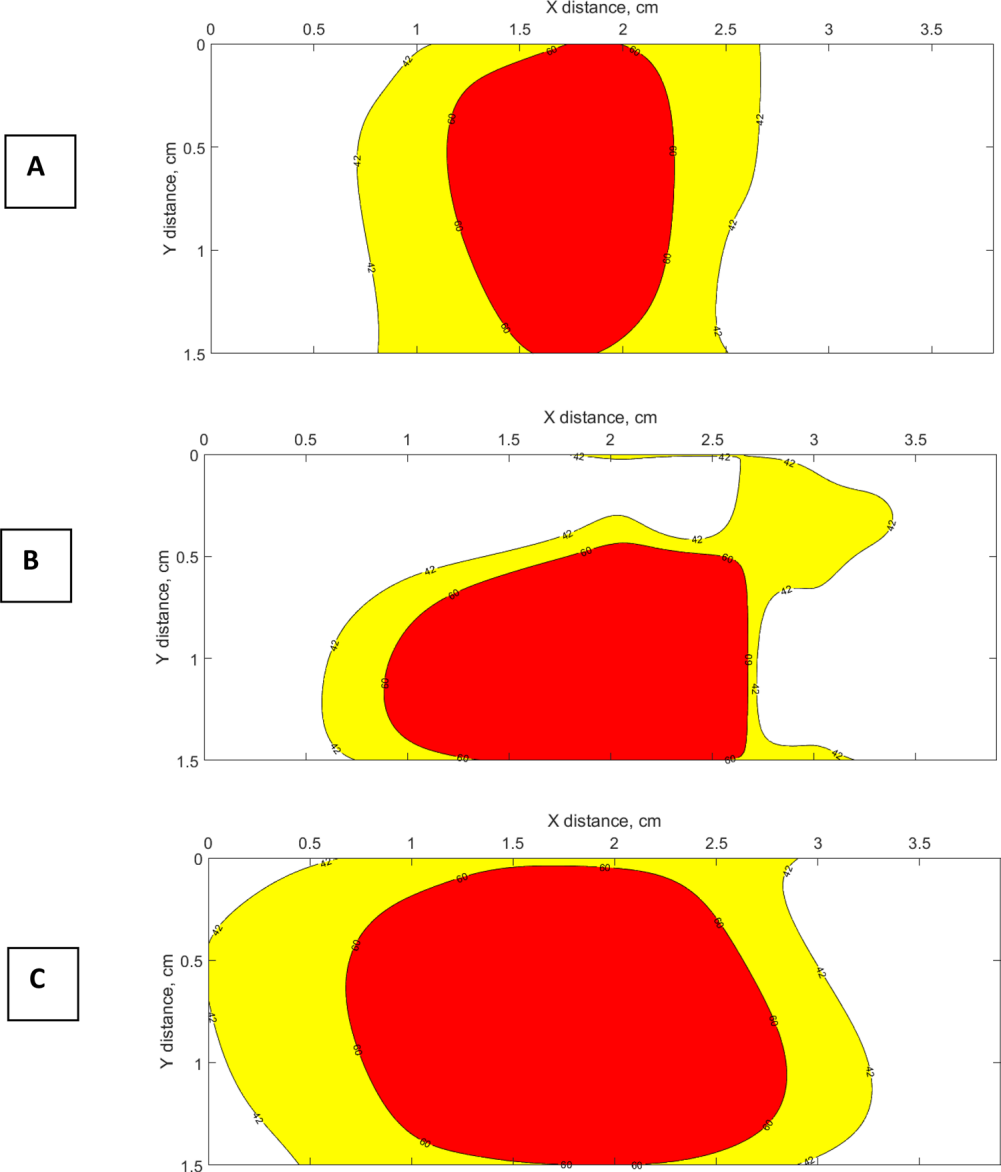
The 2D plot contours of heat increase during laser ablation at temperatures between 42 and 60 °C for liver phantom: (**A**) without nanomaterials treatment; (**B**) treated with iron oxide magnetic nanoparticles; (**C**) treated with gold nanoparticles.

According to the thermal dosimetry studies done by Sapareto and Dewey, the effect of high temperatures between 42 and 60 °C is required to conduct thermal therapy^37^. At the temperature of 42 °C the cytotoxicity occurs, while protein coagulation happens at 60 °C^38^. While the temperatures higher than 100 °C leads to unwanted results due to tissue vaporization and carbonization. Hence, the temperature range between 60 and 100 °C is effective in order to obtain the maximum ablation area. Figure 10 shows the contour plot of heat increase between 42 and 60 °C for dry tissue and tissues treated with iron oxide magnetic and gold nanoparticles.

According to the estimated area of ablation lesion (Table 1) of porcine liver without nanomaterials treatment and with the tissue injected with nanoparticles, the biggest ablated area was recorded by NPDF fibers when the liver was treated with gold nanoparticles.

The rapid temperature increment is undesirable in order to avoid tissue carbonization and vaporization which can lead to the changes of the optical properties of tissue and laser beam propagation into tissue^39^. The tissue carbonization occurs at temperatures higher than 100 °C^17^. Hence, the ability to observe the ablation process by monitoring in real-time allows stopping the experiment at the desired rate of heating. Adding to, the LA employing gold nanoparticles displayed the best scenario of gradual heating with maximum ablation area by not reaching the 100 °C.

## Discussion

Laser ablation is gaining great interest in the treatment of cancer in cases when the surgery cannot be applied due to the hard-to-reach location of the tumor or the presence of several tumors. Moreover, thermal ablation offers many advantages compared to surgical resection, which is reduced morbidity, low cost, the ability to avoid the damage of healthy tissue^40^. However, the current LA procedures have a limitation in the size of the ablation area of 1–2 cm^2^ due to the easily scattering and absorption properties of laser light affecting the reduced tissue penetration^41^. To overcome this limitation, some authors proposed the simultaneous use of multiple applicators which can increase the invasiveness of the procedure^42^. Another drawback of thermal therapies is in the failure to distinguish between damaged and healthy cells^19^. Finally, existing laser ablation technologies to treat the tumor have difficulties in efficient and real-time monitoring of applied heat, particularly in deep regions^17^.

The proposed method allowed us to monitor extensively and accurately the temperature and increase the ablation area during LA using high-scattering NPDF fibers and nanoparticles. The SLMux approach used in our LA demonstrated its effectiveness in measuring the temperature pattern in the 2-dimensional XY plane in real-time. Thus, the quality of the 2D sensing setup can overcome the existing limitations in measuring the temperature patterns. The spatial resolution of the proposed method allows us to obtain accurate information about the temperature distribution on the organ surface and is comparable with the performances of MR thermometry, considered as the gold standard. Indeed the Proton Shift Resonance method, the most common technique for estimating tissue temperature from MRI, has a spatial resolution of about 3–4 mm, which approaches the requirements for an adequate three-dimensional reconstruction of the temperature map of the heated target^43^.

The combination of LA and nanomaterials lead to a significant increase in temperature value according to the results, summarized in Table 1. Two types of nanoparticles demonstrated different temperature patterns, where gold nanoparticles distinguished the best performance showing the symmetrical round shape heating and high temperature over 100 °C, and almost double increase of the treated area. This is a 2D increase, if we extend it to 3D it means that we would obtain a 2.8 × increase of treated volume, only through the use of nanoparticles. While magnetic iron oxide nanoparticles showed a rapid increase in temperature up to 160 °C and asymmetrical heating shape. Hence, the ablation using magnetic iron oxide nanoparticles leads to a rapid increase in the temperature, while marginally increasing the treated area. That is contrary to the desired trend.

Moreover, this setup is adequate to measure the thermal patterns in LA comparing to other methods, such as MRI. Noteworthy that this setup offers the same real-monitoring opportunities, but without radiation which is the drawback of MRI.

## Conclusions

The proposed advanced technique allowed monitoring the temperature using a distributed sensing method (SLMux network) during nanoparticle-mediated laser ablation. The study validated the possibility of accurate temperature monitoring at several points in real time during LA.

In the proposed setup, we have 4 separate fibers for sensing, each suitable to be percutaneously inserted into the tissue. Hence, this setup can be suitable for operating in vivo, and is suitable for the design of a device that incorporates the laser delivery and the sensing fibers. In addition, the high scattering level of the fibers allows tagging with precision each sensing region, hence the thermal data can be aligned with great accuracy—whereas for example FBG arrays or a single distributed SMF fiber are more susceptible to misalignments of the sensing region.

Moreover, nanoparticles deposited in the tissue demonstrated the increase of ablation area by the ability to randomize the light path and improve the heating pattern. The application of nanoparticles during LA had a significant impact not only on the increase of heating area but also on the temperature elevation compared to the cases when the liver phantom was bare and not treated with nanoparticle solution. The obtained thermal maps and cytotoxicity evaluations are real-time functions, that allow to adjust the ablation time and terminate the process regarding the size of the ablated area.

However, this method has a drawback that the laser light was not introduced into the tissue interstitially. Adding to, the outcome of the LA is different over the ablation procedures limiting the repeatability of the experiments. This is governed by the different properties (optical, and thermo-optical) of the tissue, even on the same phantom; different fat percentage or collagen lead to different outcomes, even maintaining the same laser properties. This is why sensing the temperature is so essential for this type of treatment, and the effect is amplified by the presence of nanoparticles, which also affect the outcome.

Nevertheless, the obtained results could mimic the laser ablation performance on liver tissue assisted with nanomaterials. The experiment output can be extended and exploited further in clinical trials.

## Supplementary information

Supplementary video 1.

Supplementary video 2.

Supplementary video 3.
